# Aetiology of influenza-like illnesses in the pre-COVID-19 season 2019-2020: role of coronaviruses: Irene Amoruso

**DOI:** 10.1093/eurpub/ckac129.596

**Published:** 2022-10-25

**Authors:** I Amoruso, M Fonzo, C Bertoncello, V Baldo, T Baldovin

**Affiliations:** Hygiene and Public Health Unit, DCTVSP, University, Padua, Italy

## Abstract

**Background:**

Only a proportion of seasonal influenza-like illnesses (ILIs) can de facto be attributed to influenza viruses. This study investigated the aetiology of ILIs to estimate the prevalence of human coronaviruses (CoVs) and to analyse their clinical-epidemiological traits.

**Methods:**

A sample of 613 outpatients (253 adults, 360 children) with ILI in Veneto Region, Italy, was included. ILI was defined according with the EU Decision 2018/945. Sigma-Virocult nasopharyngeal swab were used. Nucleic acids were extracted with the QiaAmp Viral RNA Mini Kit (Qiagen). Molecular detection of respiratory viruses was performed with commercial One-step RT qPCR reagents (Allplex® Respiratory Panels, Seegene). Information on age, sex, symptoms, co-infections and comorbidities was collected.

**Results:**

CoVs were the 3rd most frequent pathogen in adults (7.5%, after influenza and rhinovirus) and the 4th in children (4.7%, after influenza, rhino- and adenovirus). Subtype distribution was similar, with OC43 the most frequent. Probability of CoV involvement was twice in males (AOR=2.16; 95%CI: 1.05-4.39), whereas no association with age was noted. Co-infection with other viruses was frequent in children (65% of cases). CoV symptoms were not peculiar, although respiratory tract involvement was less likely than influenza (AOR=0.13; 95%CI: 0.04-0.41). Among CoV outpatients, 36% had one or more chronic diseases, compared with 5.6% among influenza (p = 0.001).

**Conclusions:**

Even before the COVID-19 pandemic, CoVs had a substantial role in ILI aetiology: 1 case of CoV every 3 influenza infections in adults. The higher prevalence of comorbidities among CoV positives compared to influenza indirectly shows the benefits of flu vaccines in individuals at higher risk. Careful surveillance of the viruses responsible for ILI continues to be desirable, including, but not limited to, detecting a possible change in the aetiology of ILI after the administration of SARS-CoV-2 vaccines in the population.

**Key messages:**

• Pre-pandemic virological surveillance of influenza-like illnesses (ILIs) reveals how seasonal coronaviruses were the third most frequent respiratory pathogen in adults.

• Prevalence of comorbidities was significantly higher in patients with a coronavirus-related ILI compared to influenza, supporting the benefits of flu vaccination for high risk groups.

